# Chester County Medical Society

**Published:** 1847-08

**Authors:** Wilmer Worthington


### RECORD OF MEDICAL SCIENCE.

We received the following account of the proceedings of the Chester County Medical Society, from the Secretary, Dr. Worthington, in time for our last number, but by accident it was omitted. We shall be more careful of any favors we may receive from the worthy Secretary, or any of his fellow members, in future. Among the members of the Chester County Medical Society we discover the names of gentlemen who have long been eminent as practitioners, and some who have made important contributions to the literature of our profession, and we regard their re-association, aided and incited by the younger brethren to whom they have extended the right hand of fellowship, as fortunate for themselves and auspicious for science.

### CHESTER COUNTY MEDICAL SOCIETY.

The Chester County Medical Society, which had suspended its operations for sixteen years, held a meeting on the 8th of June, 1847. Members present: Dr. Wm. Darlington, President; Doctors Isaac Thomas, John B. Brinton, C. W. Parish, Isaac Pennington, and Wilmer Worthington.

The following physicians were elected members and signed the constitution :--

Doctors J. R. Walker, S. H. Harry, C. R. Parke, John Edge, W. W. Reese, E. F. Rivinus, W. D. Hartman. R. H. Smith, N. H. Clarke, John Kinsey, S. Harris, J. T. Huddleson, and E. Harvey.

The Society appointed Drs. Worthington, Parish and Rivinus to revise the constitution, and report to next stated meeting.

The following are the officers for the present year :—

*President.*—Dr. William Darlington.

1*st Vice President.*—Samuel McCleane.

2*nd Vice President.*—Isaac Thomas.

*Corresponding Secretary.*—C. W. Parish.

*Recording Secretary.*—Wilmer Worthington.

*Treasurer.*—E. F. Rivinus.

*Orator.*—Wilmer Worthington.

Drs. Pennington, Thomas, Harry, Harris, Walker, Parish, and Huddleson, were appointed a committee to ascertain and report to next stated meeting, the names and residences of all the regularly educated physicians in Chester and Delaware counties.

Adjourned to meet at Westchester, on the first Wednesday of September next, at 2 o'clock, P. M.

WILMER WORTHINGTON.

